# A case of prosthetic hip infection and abscess caused by *Trueperella bernardiae*

**DOI:** 10.1016/j.nmni.2021.100885

**Published:** 2021-04-23

**Authors:** W. Tang, K. Desai

**Affiliations:** 1) Department of Medicine, Kettering Medical Center, Kettering, OH, USA; 2) Department of Internal Medicine, Boonshoft School of Medicine, Wright State University, Dayton, OH, USA; 3) Internal Medicine & Infectious Disease Medical Director Employee Health, Kettering Health Network, Dayton, OH, USA

**Keywords:** Hip abscess, prosthetic hip infection, septic arthritis, *Trueperella bernardiae*

## Abstract

*Trueperella bernardiae* is a skin flora organism with few reported cases of pathology. Most cases have been described in urinary tract infections and skin and soft-tissue infections. We present the first known case of *T. bernardiae* as a causative agent of a prosthetic hip infection with subsequent hip abscess.

## Introduction

*Trueperella bernardiae* is a non-spore-forming, non-motile, facultative anaerobic, Gram-positive coccobacillus [1]. It is catalase-negative, with colonies that are circular, convex and smooth in appearance [1]. This organism was originally placed in the genus *Actinomyces*, then moved to the genus *Arcanobacterium*, before being reclassified into its own genus in 2011 [2]. To date, there have been only a few cases reported in the literature, ranging from urinary tract infections, knee infections, skin and soft-tissue infections and bloodstream infections [3]. These bacteria have been identified as part of the regular skin flora, and were probably dismissed as contaminants in cultures before the advent of matrix-assisted desorption–ionization time-of-flight mass spectrometry (MALDI-TOF MS) [4]. Furthermore, these organisms do not grow well on plated media, leading to false-negative culture results [5]. We report a rare case of a prosthetic hip joint infection caused by this pathogen. To the best of our knowledge, *T. bernardiae* has never been reported to have been the causative agent of a hip abscess.

## Case description

**Fig. 1:**
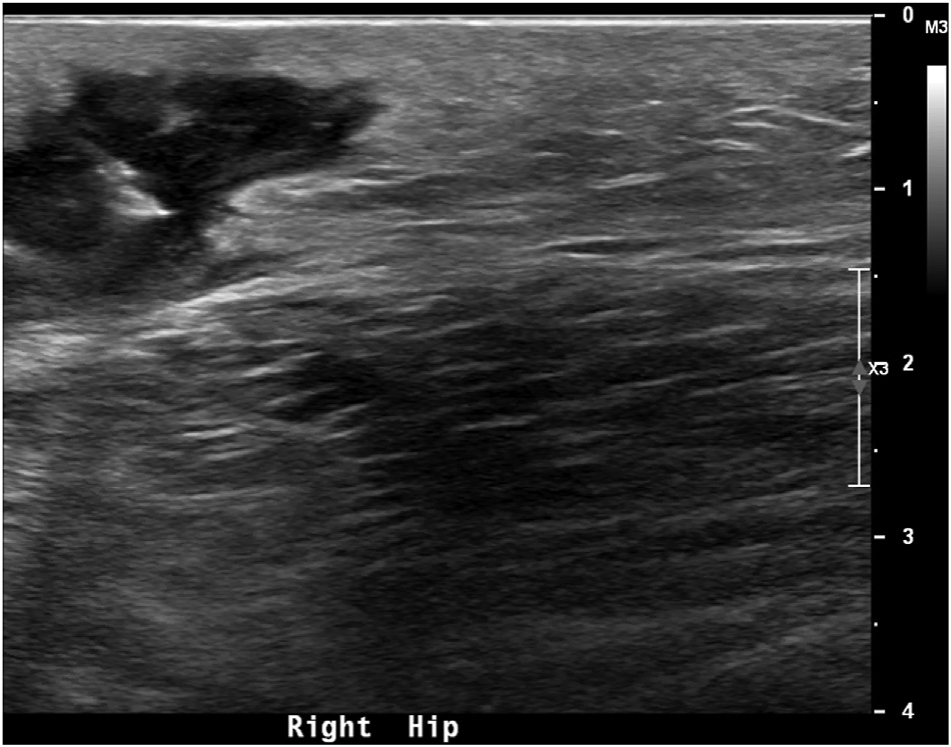
Ultrasound imaging of the right hip showing a complex fluid collection suggestive of haematoma or abscess.

A 71-year-old otherwise healthy man presented to the emergency department with a bulge on his right hip at an area of surgical scar from a total hip arthroplasty performed in October 2018 (2 years before presentation). He first noticed a bulge around the surgical scar 6 weeks before arrival and had undergone an ultrasound scan of the hip 1 week before presentation on an outpatient basis. This showed a complex fluid collection measuring 9.1 × 3.7 × 3.2 cm with a volume of 59 mL suggestive of a haematoma (Fig. 1). At the time, only 1 mL of blood-tinged fluid could be aspirated. Using aerobic cultures and MALDI-TOF MS, the organism would later be identified as *T. bernardiae.* No *in vitro* susceptibility testing was performed, and the patient was not treated with antibiotics. His right hip bulge did not resolve with aspiration, and increased in size along with new symptoms of pain, tenderness, warmth and redness prompting presentation to the emergency department. He denied fever, chills, nausea and vomiting. On this visit to the emergency department, an 18-gauge needle was inserted into the centre of greatest fluctuance and 4.5 mL of seropurulent material was aspirated. He was given a regimen of doxycycline for 7 days and told to follow up with his orthopedic surgeon as soon as possible.

**Fig. 2:**
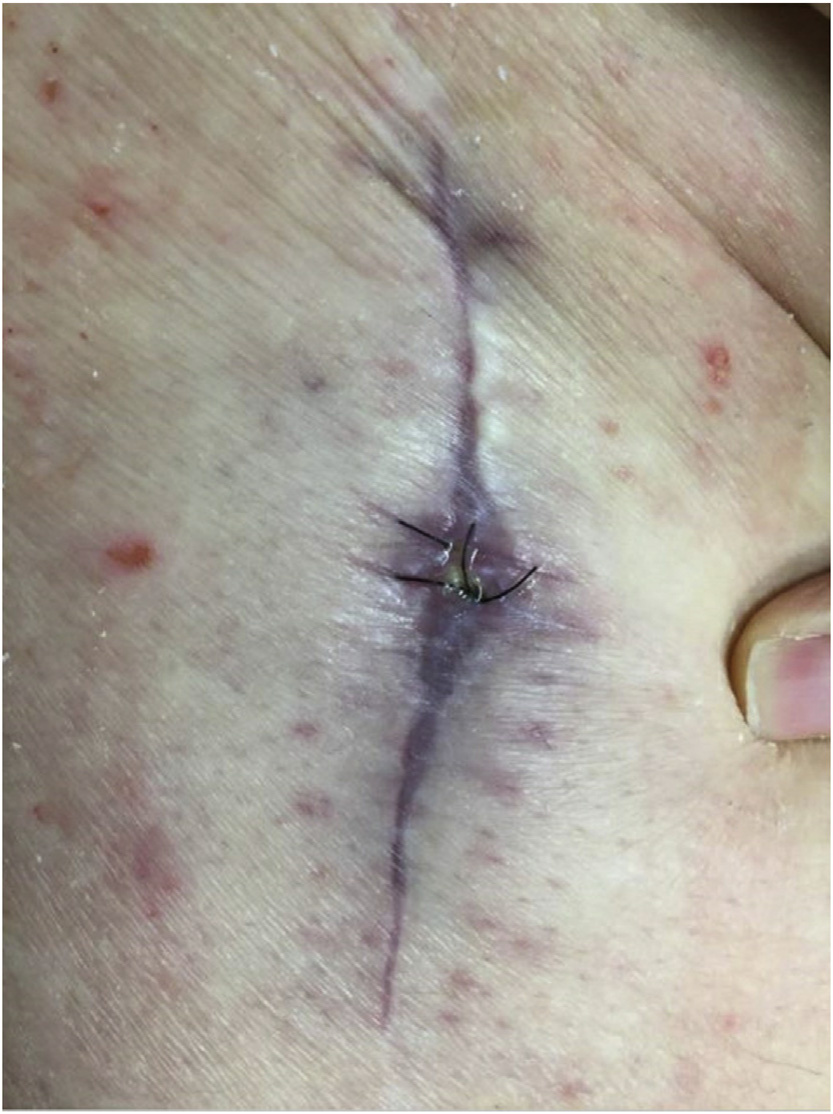
Image of right hip wound with continued slow drainage without erythema or pain, soaking through pressure dressings.

The patient presented to his orthopedic surgeon 3 days later, by which time cultures from the aspiration with the assistance of MALDI-TOF MS technology were positive for *T. bernardiae*. Again, no *in vitro* susceptibility testing was performed. During this evaluation, it was noted that there was persistent drainage from the abscessed area, not previously present, and probably the result of previous aspiration and tract formation from the needle. Incision and drainage, washout, polyethylene exchange, head exchange, along with placement of antibiotic beads were considered and offered to the patient. The patient was amenable to the plan, and underwent outpatient surgery 8 days after initial presentation to the emergency department. During surgery, there was no fluid from the wound, no abscess was appreciated, and the prosthesis was found to be without defect or effusion. The decision was made to leave the prosthesis in place. Three litres of pulse lavage was performed and Stimulan® beads (Biocomposites Inc., 700 Military Cutoff Road, Suite 320, Wilmington, NC 28405, USA) with vancomycin were placed into made tracks. Surgical cultures were obtained by swabbing fatty and soft tissue around the prosthesis. For an additional 2 weeks post-surgery, the patient had continued drainage without erythema or pain, soaking through pressure dressings (Fig. 2).

Four weeks after the incision and drainage, the patient was then referred by the orthopedic surgeon to a plastic surgeon and wound specialist who also observed continuous serosanguinous fluid drainage, thought to be from a seroma rather than a deep space infection. Sharp excisional debridement and wound VAC placement was recommended and was performed 2.5 months after the initial emergency department visit, with additional cultures taken. Isolates returned positive as the same organisms from the original visit to the emergency department, using similar identification methods. An infectious disease specialist was then consulted and it was felt that, as the infection probably extended up to the prosthetic joint, the infection should be treated aggressively with intravenous antibiotics followed by chronic suppressive antibiotics. The patient agreed, and a peripherally inserted central catheter line was placed for 6 weeks of intravenous ceftriaxone. The patient has been symptom free since completion of treatment and his wound has been healing well. He has been placed on chronic suppressive cefadroxil with a plan to continue for at least 1–2 years.

## Discussion

The incidence of *T. bernardiae* infections to date is largely unknown, because the organism was probably misclassified as other bacteria including coryneform bacteria, Gram-positive bacilli or streptococci before the widespread usage of MALDI-TOF MS [6]. Furthermore, because the organism is recognized as part of normal skin flora, it may not have been given the attention warranting that of a human pathogen [4]. In our literature review, we have only found a handful of cases of *Trueperella* infections causing human pathology including bacteraemia, wound infections, septic arthritis and brain abscesses [[1], [2], [3],[6], [7], [8], [9]]. In particular, we have found only one case implicating *T. bernardiae* as the cause of a prosthetic knee infection [10]. The present case adds to the literature as being the first known case to demonstrate a prosthetic hip infection caused by *T. bernardiae.*

*In vitro* susceptibility has included sensitivity to β-lactams, clindamycin and vancomycin, with resistance to ciprofloxacin, aminoglycosides and metronidazole reported [7,11,12]. Otto *et al.* [4] successfully treated a *Trueperella* infection with amoxicillin/clavulanate, while Rattes *et al.* [1] demonstrated success with piperacillin/tazobactam and vancomycin. However, treatment guidelines for these microorganisms have not been established because of the scarcity of data. In our case, it is unclear if the organism was inoculated at the time of original surgery or not. His clinical course is suggestive of a lower virulence of this organism. The patient was successfully treated with intravenous ceftriaxone for 6 weeks, without recurrence.

In summary, we report a case of *T. bernardiae* as a causative agent for a septic hip with an associated abscess, in an otherwise healthy adult with previous total hip arthroplasty 2 years before. To the authors' knowledge, this is the first case where this organism has been recognized as a causative agent of a prosthetic joint infection and successfully treated. This case highlights the need to further characterize this organism, because it may have greater pathogenic potential than previously recognized, and to further elucidate appropriate antimicrobial therapy.

## Conflicts of interest

The authors declare that they have no conflicts of interest.

## Author contributions

WT and KD designed and conducted the research. KD provided the data. WT had primary responsibility for final content. Both authors read and approved the final manuscript.
